# Interaction between the Gut Microbiota and Intestinal Motility

**DOI:** 10.1155/2022/3240573

**Published:** 2022-11-15

**Authors:** Qihong Liu, Yunfeng Luo, Xiao Ke

**Affiliations:** ^1^The Second People's Hospital Affiliated to Fujian University of Chinese Medicine, Fuzhou 350003, China; ^2^Fujian Clinical Medical Research Centre of Chinese Medicine for Spleen and Stomach, Fuzhou 350003, China

## Abstract

The gut microbiota is the largest symbiotic ecosystem with the host and has been proven to play an important role in maintaining the stability of the intestinal environment. The imbalance of the gut microbiota is caused by the imbalance between the symbiotic microbiota and the pathogenic microbiota. The commensal microbiome regulates intestinal motility, while the pathogenic microbiome causes intestinal motility disorder, resulting in disease development. Intestinal motility is a relatively general term, and its meaning may include intestinal muscle contraction, intestinal wall biomechanics, intestinal compliance, and transmission. The role of intestinal microecology and intestinal motility are interrelated, intestinal flora disorder mediates intestinal motility, and abnormal intestinal motility affects colonization of the intestinal flora. In this review, we briefly outlined the interaction between gut microbiota and intestinal motility and provided a reference for future studies.

## 1. Introduction

### 1.1. Gut Microbiota

The intestine of mammals contains a microbial community, defined as the microbiome, including bacteria, viruses, and fungi. The microbial genome sequence contains 3 × 10^6^ genes, which is about 150 times the length of the human genome [1]. Gut microbiota (GM) includes 1000 to 1500 types of bacteria; however, a person contains only approximately 160 bacterial species, which indicates that there are substantial differences in the composition of the microbiome between individuals [2]. In recent decades, next-generation sequence technologies have contributed to understanding the complex relationship between the microbiome and related diseases. 16Sr RNA sequence results show that *Firmicutes* and *Bacteroidetes* account for approximately 92% of the human GM [3]. The distribution and composition of the intestinal flora often vary among individuals based on their age [4], physical condition [5], diet [6], and living environment [7]. Generally, the relationship between the intestinal flora and the host is dynamic and relatively harmonious [8]. The GM plays an important role in protecting the host of pathogenic microbes, modulating immunity, and regulating metabolic processes, and is even regarded as an endocrine organ [9]. The symbiotic relationship between the GM and the host is mutually beneficial. The host provides important habitat and nutrients for the GM. The GM is not evenly distributed among the gastrointestinal tract, and there are certain differences in the type, distribution, and quantity of the microbiota [10]. The number of bacteria gradually increases from top to bottom from the direction of the digestive tract, with the largest number in the colon [11]. The GM supports the development of the metabolic system and maintains normal intestinal motility by providing beneficial nutrients, such as the synthesis of vitamins and short-chain fatty acids (SCFAs) [12, 13]. Hence, the interaction between the microbiome and intestinal motility system is essential to maintain the normal function of intestinal motility.

### 1.2. Intestinal Motility

“Intestinal motility” is a more general term, which may include intestinal muscle contraction movement, intestinal wall biomechanics (tension movement), intestinal cavity compliance and related activities, and transmission [14–16]. The contraction activity of intestinal smooth muscle cells is called slow-wave activity, which is also a temporal (short) contraction [17]. Intestinal tension movement (long-term contraction) usually occurs after eating and is regulated by mechanical or neural stimulation [18]. Intestinal compliance refers to the ability of the intestine to locally adapt to the expansion of the cavity, which is the ratio of volume changes to pressure changes [19]. Transmission refers to the time it takes for food or other substances to pass through a specific area of the intestine, reflecting the overall result of the interaction of a variety of intricate parameters (contraction activity, tension, compliance, etc.) [20]. In addition to the realization of propulsive movement, the motor function of the colon also includes receptivity or storage and rapid emptying during defecation. The main modes of propulsive movement include intestinal migrating motor complexes (CMMCs), high amplitude propagated contractions (HAPCs), and giant migration contraction (GMCs) [21]. Intestinal motility is mainly regulated by the neuroendocrine system, which consists of 3 levels [22]: local regulation of the enteric nervous system (ENS); regulation of prevertebral ganglia on the enteric nervous system and central nervous system information; the central nervous system passes through the autonomic nervous system and the neuroendocrine system, which is also called the brain-gut axis [23]. The regulation of intestinal function or power through this biological axis is called brain-gut or gut-brain interactions [24]. Among them, the enteric nervous system is the most important, followed by the autonomic nervous system and the enteric nervous system. At the same time, GM is also involved in the regulation of intestinal motility [25]. In the past ten years, a large number of studies have confirmed that functional bowel disease is accompanied by GM imbalance [26].

## 2. Gut Microbiome and Intestinal Motility

### 2.1. The Dysbiosis of the GM Induces Intestinal Motility Disorder

#### 2.1.1. GM Influences ENS Development

The GM is very important for the development and homeostasis of the ENS. Germ-free (GF) animals are an ideal model to explore the relationship between each other [27]. The number of enteric neurons in GF mice is reduced [28], the development of enteric glial cells (EGCs) is abnormal [29], and the excitability of intrinsic primary afferent neurons is weakened [30], which leads to defects in the assembly of enteric nerve circuits and the signal of the brain-gut axis, showing the time of gastric emptying and intestinal transit Prolongation, intestinal motility disorder [31]. Some studies have found that the network density of EGCs of GF mice is restored and the intestinal motility is improved after administration of normal flora [32, 33]. As far as we know, the GF mice model has several limitations as GF mice are born in aseptic conditions, which may not only affect the development of the gut motility system, but also the immunity system and metabolic function [34]. Therefore, some studies have used antibiotics to eliminate the intestinal flora to establish a pseudogerm-free (PGF) model to verify whether the effect of the microbiota on intestinal motility is reversible. Ge et al. [35] showed that PGF mice delayed gastrointestinal and colonic motility, and inhibition of phasic contractions of longitudinal muscle from the isolated proximal colon. Besides, serotonin, tryptophan hydroxylase 1, and secondary bile acids levels were decreased.

#### 2.1.2. Changes in the Structure of the GM Affect Intestinal Motility

Mainly present with symptoms dominated by the gastrointestinal tract, such as constipation, diarrhea, and abdominal distension. The results of several adult constipation studies are quite consistent. In patients with functional constipation and constipation-type irritable bowel syndrome, there is a decrease in *Bifidobacterium* and *Lactobacillus* and an increase in Bacteroides [36, 37]. Researchers supplemented with probiotics VSL#3 (4 types of *Lactobacilli* + 3 types of *Bifidobacteria* + 1 type of *Streptococcus thermophilus*), which can effectively improve the frequency and characteristics of bowel movements and relieve symptoms of constipation [38]. The researchers implanted the fecal flora of STC patients into PGF mice to induce the latter to produce STC symptoms and accompanied by changes in the structure of the flora, in which the abundance of ecomania was significantly increased [39]. In recent years, it has been discovered that the intestinal flora can communicate with the brain-gut axis in two directions to regulate the movement and sensory function of the colon, and the concept of the “microbiota-brain-gut axis” has emerged [40].

#### 2.1.3. Metabolites of the GM Affect Intestinal Dynamics

Short-chain fatty acids (SCFAs) are the metabolites of dietary fiber fermented by a type of bacteria, mainly acetic acid, propionic acid, and butyric acid [41, 42]. SCFAs are nutrients for intestinal epithelial cells and are of great significance to host physiology [43]. SCFAs have anti-inflammatory [44], anticancer [45], intestinal barrier enhancement [46], and promotility effects [47]. The mechanism of SCFAs regulating intestinal motility is as follows: (i) SCFAs can upregulate the expression of TPH1, thereby promoting 5-HT synthesis [48]. (ii) SCFAs activate *G*protein-coupled receptors such as GPR41 and GPR43 and mediate the secretion of glucagon-likepeptide-1 (GLP-1), thereby regulating gastrointestinal motility [49]. (iii) Butyrate combined with monocarboxylic acid transporter (MCT) 2, increases the ratio of intermuscular cholinergic neurons, adjusts the neurochemical phenotype of ENS, enhances the contraction of the colonic circular muscle, and speeds up transmission [50]. (iv) Butyrate has biphasic effects on colonic motility, it stimulates motility at low and inhibits motility at higher concentrations [51, 52]. In addition, bile acid hydrolase secreted by certain intestinal flora can metabolize bile acids produced by the liver into unconjugated bile acids, and it can promote intestinal motility and induce bile acid-related diarrhea under certain circumstances [52]. The gas produced by the intestinal flora metabolizing substrates, such as methane, can also affect the contraction of intestinal smooth muscle and slow down the transmission [53].

### 2.2. The Abnormal Intestinal Motility Affects GM

#### 2.2.1. “*r*/*K* Selection” Theory and GM

Normal intestinal motility is an important means to prevent the imbalance of intestinal flora. Normal movement in the digestive and interdigestive periods of the intestine can remove bacteria [54]. Studies have shown that intestinal motility disorders are mainly manifested in the colon, but there are also certain degrees of kinetic abnormalities in the small intestine and rectum [55]. The human intestine is a diverse ecosystem, containing trillions of microbial cells and hundreds of microbial species. Like other ecosystems, the intestinal flora can respond to changes in surrounding environmental factors, so abnormal intestinal motility will affect the composition and function of the microbiota. This view is in line with the “r/K selection” theory of environmental disturbance in ecology, which was proposed by the famous ecologists MacArthur and Wilson in 1967 on the life history strategy of “population reproduction” [56]. The “*r*/*K* choice” theory means that organisms will choose a suitable way of survival for their different living conditions. For example, when the intestinal transit is accelerated and the competitiveness of the intestinal environment is reduced, the bacteria with high fertility can better adapt to growth, which is called “r-selection”. On the contrary, when the intestinal transit time slows down and the surrounding competitive environment intensifies, high viability bacteria can grow slowly under unrestricted conditions, which is called “*K*-selection”. Therefore, the acceleration or slowdown of intestinal motility will affect the reproduction of suitable bacterial species [57].

#### 2.2.2. Faster or Slower Gut Motility Selects Survival of Suitable GM

Normal intestinal motility is an important way to prevent intestinal dysbiosis, and normal intestinal motility during the digestive and interdigitate phases have a role in the removal of bacteria. Intestinal motility disorders are mainly characterized by accelerated or slowed motility, the most common clinical symptoms such as diarrhea or constipation. Just take, for instance, patients of irritable bowel syndrome with diarrhea (IBS-D), it was found that the abundance of *Prevotella*, *Shigella*, and *Enterobacter* was increased, while the abundance of Bifidobacterium, *Bacteroides*, and *Lactobacillus* was decreased [58]. In patients of irritable bowel syndrome with constipation (IBS-D), the abundance of *Bacteroides*, *Enterobacte*r, and *Eubacterium* increased, and the abundance of *Bifidobacterium*, *Faebacterium*, and *Prevotella* decreased [59]. In patients with slow transit constipation (STC), the abundance of *Veillonella*, *Faebacterium*, *Enterobacter*, and *Escherichia coli* was increased, while the abundance of *Prevotella*, Bifidobacterium, *Bacteroides*, and *Bacillus* spp. degree reduction [60]. It is not difficult to see that the abundance of *Prevotella* increases in patients with diarrhea and decreases in patients with constipation; the abundance of beneficial bacteria such as *Bifidobacterium*, *Bacteroides*, and *Lactobacillus* decreases in patients with diarrhea and constipation. Therefore, the abnormality of intestinal motility will have a direct impact on specific groups in the microbiota and cause significant changes in the intestinal ecosystem.

**Figure 1:**
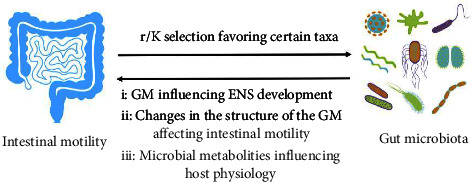
Model for interactions between gut microbiota and intestinal motility.

In conclusion, the roles of gut flora and gut dynamics are interrelated. Dysbiosis mediates intestinal dynamics and abnormal intestinal dynamics affect intestinal population reproduction, and a model of the interaction between the two is shown in Figure 1.

## 3. Conclusions

In summary, intestinal microbes and their metabolites help maintain normal intestinal motility, intestinal motility affects the growth and reproduction of intestinal flora. Recent data have shown the pivotal role of intestinal microbiota in intestinal motility. An abnormal interaction between GM and intestinal motility is associated with the pathogenesis of intestinal motility disorders diseases, such as IBD-C, IBD-D, and STC, and it highlights the importance of exploring the function of microbiota in such diseases. Thus, GM has become an effective target for the development of new diagnostic methods. Balancing the GM will likely represent an effective treatment for intestinal motility disorders diseases. At the same time, the understanding of the relationship between the GM and intestinal motility needs to be improved, and further discussions about these mechanisms will be of great help in guiding clinical practices.

## Data Availability

No underlying data was collected or produced in this report.

## References

[1] Lepage P., Leclerc M. C., Joossens M. (2013). A metagenomic insight into our gut’s microbiome. *Gut*.

[2] Wang W. L., Xu S. Y., Ren Z. G., Tao L., Jiang J. W., Zheng S. S. (2015). Application of metagenomics in the human gut microbiome. *World Journal of Gastroenterology*.

[3] Eckburg P. B., Bik E. M., Bernstein C. N. (2005). Diversity of the human intestinal microbial flora. *Science*.

[4] Maynard C., Weinkove D. (2018). The gut microbiota and ageing. *Subcellular Biochemistry*.

[5] Codella R., Luzi L., Terruzzi I. (2018). Exercise has the guts: how physical activity may positively modulate gut microbiota in chronic and immune-based diseases. *Digestive and Liver Disease*.

[6] Hills R. D., Pontefract B. A., Mishcon H. R., Black C. A., Sutton S. C., Theberge C. R. (2019). Gut microbiome: profound implications for diet and disease. *Nutrients*.

[7] Winglee K., Howard A. G., Sha W. (2017). Recent urbanization in China is correlated with a Westernized microbiome encoding increased virulence and antibiotic resistance genes. *Microbiome*.

[8] Rumney C. J., Rowland I. R. (1992). In vivo and in vitro models of the human colonic flora. *Critical Reviews in Food Science and Nutrition*.

[9] Possemiers S., Bolca S., Verstraete W., Heyerick A. (2011). The intestinal microbiome: a separate organ inside the body with the metabolic potential to influence the bioactivity of botanicals. *Fitoterapia*.

[10] Martinez-Guryn K., Leone V., Chang E. B. (2019). Regional diversity of the gastrointestinal microbiome. *Cell Host & Microbe*.

[11] Zeng H., Umar S., Rust B., Lazarova D., Bordonaro M. (2019). Secondary bile acids and short chain fatty acids in the colon: a focus on colonic microbiome, cell proliferation, inflammation, and cancer. *International Journal of Molecular Sciences*.

[12] Riccio P., Rossano R. (2018). Diet, gut microbiota, and vitamins D + A in multiple sclerosis. *Neurotherapeutics*.

[13] Parada Venegas D., De la Fuente M. K., Landskron G. (2019). Short chain fatty acids (SCFAs)-Mediated gut epithelial and immune regulation and its relevance for inflammatory bowel diseases. *Frontiers in Immunology*.

[14] Sarna S. K. (1993). Colonic motor activity. *Surgical Clinics of North America*.

[15] Siri S., Zhao Y., Maier F., Pierce D. M., Feng B. (2020). The macro- and micro-mechanics of the colon and rectum I: experimental evidence. *Bioengineering*.

[16] Van Daele E., Knol J., Belzer C. (2019). Microbial transmission from mother to child: improving infant intestinal microbiota development by identifying the obstacles. *Critical Reviews in Microbiology*.

[17] Rychter J., Espín F., Gallego D., Vergara P., Jiménez M., Clavé P. (2014). Colonic smooth muscle cells and colonic motility patterns as a target for irritable bowel syndrome therapy: mechanisms of action of otilonium bromide. *Therap Adv Gastroenterol*.

[18] Cole S. J., Duncan H. D., Silk D. B. A. (1998). Intestinal motility. *Current Opinion in Clinical Nutrition and Metabolic Care*.

[19] Mouchli M. A., Camilleri M., Lee T. (2016). Evaluating the safety and the effects on colonic compliance of neostigmine during motility testing in patients with chronic constipation. *Neuro-Gastroenterology and Motility*.

[20] White A. R., Werner C. M., Holmes G. M. (2020). Diminished enteric neuromuscular transmission in the distal colon following experimental spinal cord injury. *Experimental Neurology*.

[21] Dinning P. G. (2018). A new understanding of the physiology and pathophysiology of colonic motility?. *Neuro-Gastroenterology and Motility*.

[22] Bonaz B. L., Bernstein C. N. (2013). Brain-gut interactions in inflammatory bowel disease. *Gastroenterology*.

[23] O’Mahony S., Clarke G., Borre Y. E., Dinan T. G., Cryan J. F. (2015). Serotonin, tryptophan metabolism and the brain-gut-microbiome axis. *Behavioural Brain Research*.

[24] Mörkl S., Butler M. I., Holl A., Cryan J. F., Dinan T. G. (2020). Probiotics and the microbiota-gut-brain Axis: focus on psychiatry. *Current Nutrition Reports*.

[25] Mulak A., Bonaz B. (2015). Brain-gut-microbiota axis in Parkinson’s disease. *World Journal of Gastroenterology*.

[26] Clemente J. C., Manasson J., Scher J. U. (2018). The role of the gut microbiome in systemic inflammatory disease. *BMJ*.

[27] Al-Asmakh M., Zadjali F. (2015). Use of germ-free animal models in microbiota-related research. *Journal of Microbiology and Biotechnology*.

[28] Fülling C., Dinan T. G., Cryan J. F. (2019). Gut microbe to brain signaling: what happens in vagus. *Neuron*.

[29] Yu Y. B., Li Y. Q. (2014). Enteric glial cells and their role in the intestinal epithelial barrier. *World Journal of Gastroenterology*.

[30] Furness J. B., Kunze W. A., Bertrand P. P., Clerc N., Bornstein J. C. (1998). Intrinsic primary afferent neurons of the intestine. *Progress in Neurobiology*.

[31] Scarpignato C., Pelosini I. (1999). Management of irritable bowel syndrome: novel approaches to the pharmacology of gut motility. *Canadian Journal of Gastroenterology*.

[32] Kabouridis P. S., Lasrado R., McCallum S. (2015). Microbiota controls the homeostasis of glial cells in the gut lamina propria. *Neuron*.

[33] De Vadder F., Grasset E., Mannerås Holm L. (2018). Gut microbiota regulates maturation of the adult enteric nervous system via enteric serotonin networks. *Proceedings of the National Academy of Sciences of the United States of America*.

[34] Mayer E. A., Tillisch K., Gupta A. (2015). Gut/brain axis and the microbiota. *Journal of Clinical Investigation*.

[35] Ge X., Ding C., Zhao W. (2017). Antibiotics-induced depletion of mice microbiota induces changes in host serotonin biosynthesis and intestinal motility. *Journal of Translational Medicine*.

[36] Khalif I. L., Quigley E. M., Konovitch E. A., Maximova I. D. (2005). Alterations in the colonic flora and intestinal permeability and evidence of immune activation in chronic constipation. *Digestive and Liver Disease*.

[37] Parthasarathy G., Chen J., Chen X. (2016). Relationship between microbiota of the colonic mucosa vs feces and symptoms, colonic transit, and methane production in female patients with chronic constipation. *Gastroenterology*.

[38] Kim S. E., Choi S. C., Park K. S. (2015). Change of fecal flora and effectiveness of the short-term VSL#3 probiotic treatment in patients with functional constipation. *J Neurogastroenterol Motil*.

[39] Cao H., Liu X., An Y. (2017). Dysbiosis contributes to chronic constipation development via regulation of serotonin transporter in the intestine. *Scientific Reports*.

[40] de Castro A. P., de Castro A. P. (2017). Insight into role of microbiota-gut-brain peptides as a target for biotechnology innovations. *Frontiers in Bioscience*.

[41] Ratajczak W., Rył A., Mizerski A., Walczakiewicz K., Sipak O., Laszczyńska M. (2019). Immunomodulatory potential of gut microbiome-derivedshort-chain fatty acids (SCFAs). *Acta Biochimica Polonica*.

[42] Dalile B., Van Oudenhove L., Vervliet B., Verbeke K. (2019). The role of short-chain fatty acids in microbiota-gut-brain communication. *Nature Reviews Gastroenterology & Hepatology*.

[43] Tan J., McKenzie C., Potamitis M., Thorburn A. N., Mackay C. R., Macia L. (2014). The role of short-chain fatty acids in health and disease. *Advances in Immunology*.

[44] Li M., van Esch B. C. A. M., Wagenaar G. T. M., Garssen J., Folkerts G., Henricks P. A. J. (2018). Pro- and anti-inflammatory effects of short chain fatty acids on immune and endothelial cells. *European Journal of Pharmacology*.

[45] Wang G., Yu Y., Wang Y. Z. (2019). Role of SCFAs in gut microbiome and glycolysis for colorectal cancer therapy. *Journal of Cellular Physiology*.

[46] Felizardo R. J. F., Watanabe I. K. M., Dardi P., Rossoni L. V., Câmara N. O. S. (2019). The interplay among gut microbiota, hypertension and kidney diseases: the role of short-chain fatty acids. *Pharmacological Research*.

[47] Priyadarshini M., Kotlo K. U., Dudeja P. K., Layden B. T. (2018). Role of short chain fatty acid receptors in intestinal physiology and pathophysiology. *Comprehensive Physiology*.

[48] Lund M. L., Egerod K. L., Engelstoft M. S. (2018). Enterochromaffin 5-HT cells - a major target for GLP-1 and gut microbial metabolites. *Molecular Metabolism*.

[49] Ang Z., Ding J. L. (2016). GPR41 and GPR43 in obesity and inflammation - protective or causative?. *Frontiers in Immunology*.

[50] Soret R., Chevalier J., De Coppet P. (2010). Short-chain fatty acids regulate the enteric neurons and control gastrointestinal motility in rats. *Gastroenterology*.

[51] Neunlist M., Dobreva G., Schemann M. (1999). Characteristics of mucosally projecting myenteric neurones in the Guinea-pig proximal colon. *The Journal of Physiology*.

[52] Squires P. E., Rumsey R. D., Edwards C. A., Read N. W. (1992). Effect of short-chain fatty acids on contractile activity and fluid flow in rat colon in vitro. *American Journal of Physiology - Gastrointestinal and Liver Physiology*.

[53] Wolf P. G., Parthasarathy G., Chen J. (2017). Assessing the colonic microbiome, hydrogenogenic and hydrogenotrophic genes, transit and breath methane in constipation. *Neuro-Gastroenterology and Motility*.

[54] Harris L. A., Baffy N. (2017). Modulation of the gut microbiota: a focus on treatments for irritable bowel syndrome. *Postgraduate Medicine*.

[55] Deloose E., Janssen P., Depoortere I., Tack J. (2012). The migrating motor complex: control mechanisms and its role in health and disease. *Nature Reviews Gastroenterology & Hepatology*.

[56] Pianka E. R. (1970). On r and K selection. *The American Naturalist*.

[57] Cassill D. L. (2019). Extending r/K selection with a maternal risk-management model that classifies animal species into divergent natural selection categories. *Scientific Reports*.

[58] Downs I. A., Aroniadis O. C., Kelly L., Brandt L. J. (2017). Postinfection irritable bowel syndrome: the links between gastroenteritis, inflammation, the microbiome, and functional disease. *Journal of Clinical Gastroenterology*.

[59] Ohkusa T., Koido S., Nishikawa Y., Sato N. (2019). Gut microbiota and chronic constipation: a review and update. *Frontiers of Medicine*.

[60] Müller M., Hermes G. D. A., Canfora E. E. (2020). Distal colonic transit is linked to gut microbiota diversity and microbial fermentation in humans with slow colonic transit. *American Journal of Physiology-Gastrointestinal and Liver Physiology*.

